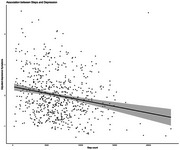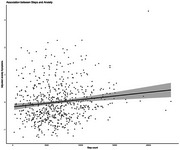# Are steps associated with mental health in older adults at risk of dementia?

**DOI:** 10.1002/alz70856_105643

**Published:** 2026-01-08

**Authors:** Yolanda Lau, Harisd Phannarus, Zuzana Walker, Claudia Cooper, Harriet Demnitz‐King, Natalie L Marchant

**Affiliations:** ^1^ Division of Psychiatry, University College London, London, United Kingdom; ^2^ Faculty of Medicine Siriraj Hospital, Mahidol University, Bangkok, Thailand; ^3^ Essex Partnership University NHS Foundation Trust, Wickford, United Kingdom; ^4^ Queen Mary University of London, London, United Kingdom; ^5^ University College London, London, United Kingdom

## Abstract

**Background:**

Depressive and anxiety symptoms are prevalent among older adults and associated with increased dementia risk. Research suggests higher step counts are associated with fewer depressive and anxiety symptoms in healthy older adults however it is unknown whether objectively measured step counts are associated with depressive and anxiety symptoms in individuals at risk for dementia (subjective cognitive decline [SCD] and mild cognitive impairment [MCI]).

**Method:**

Baseline data from 629 older adults with SCD or MCI from the APPLE‐Tree trial were utilized. Participants wore wrist‐worn wearables for two weeks, from which an average objective step count was calculated. Depressive and anxiety symptoms were assessed using the Hospital Anxiety Depression Scale. Associations between steps, depressive and anxiety symptoms were assessed separately in linear regressions adjusted for age, sex, education, and ethnicity, cognitive impairment level, anxiety symptoms (for depression model) or depressive symptoms (for anxiety model), subjective health, social support, and living arrangement. Sensitivity analyses examined whether this association differed by sex (male vs. female), cognitive impairment level (SCD vs. MCI) and symptom severity (clinical vs. non‐clinical).

**Result:**

Higher step counts were associated with fewer depressive symptoms (β =‐0.11, 95% CI: ‐0.17 to ‐0.05, *p* = 0.001), and lower likelihood of experiencing clinical levels of depressive symptoms (OR = 0.55, 95% CI 0.38 ‐ 0.79, *p* = 0.001). Higher step counts were associated with more anxiety symptoms (β=0.12, 95% CI 0.06 to 0.19, *p* = 0.003) and increased likelihood of experiencing clinical levels of anxiety symptoms (OR=1.40, 95%CI 1.09 to 1.80, *p* = 0.009). No interactions were observed with sex or cognitive impairment level in either depression or anxiety models.

**Conclusion:**

Our findings extend the literature on healthy older adults by demonstrating that higher objective steps are associated with fewer depressive symptoms in older adults at risk for dementia. However, contrary to existing evidence, we observed a positive association between step count and anxiety symptoms. Steps measured via wrist‐worn wearables could be explored as a proxy marker for depressive and anxiety symptoms, aiding early detection of these symptoms and intervention.